# Interleaved EPI Based fMRI Improved by Multiplexed Sensitivity Encoding (MUSE) and Simultaneous Multi-Band Imaging

**DOI:** 10.1371/journal.pone.0116378

**Published:** 2014-12-30

**Authors:** Hing-Chiu Chang, Pooja Gaur, Ying-hui Chou, Mei-Lan Chu, Nan-kuei Chen

**Affiliations:** 1 Brain Imaging and Analysis Center, Duke University Medical Center, Durham, NC, United States of America; 2 Department of Chemical and Physical Biology, Vanderbilt University, Nashville, TN, United States of America; 3 Vanderbilt University Institute of Imaging Science, Nashville, TN, United States of America; 4 Department of Psychiatry and Behavioral Sciences, Duke University Medical Center, Durham, NC, United States of America; 5 Graduate Institute of Biomedical Electronics and Bioinformatics, National Taiwan University, Taipei, Taiwan; National Yang-Ming University, Taiwan

## Abstract

Functional magnetic resonance imaging (fMRI) is a non-invasive and powerful imaging tool for detecting brain activities. The majority of fMRI studies are performed with single-shot echo-planar imaging (EPI) due to its high temporal resolution. Recent studies have demonstrated that, by increasing the spatial-resolution of fMRI, previously unidentified neuronal networks can be measured. However, it is challenging to improve the spatial resolution of conventional single-shot EPI based fMRI. Although multi-shot interleaved EPI is superior to single-shot EPI in terms of the improved spatial-resolution, reduced geometric distortions, and sharper point spread function (PSF), interleaved EPI based fMRI has two main limitations: 1) the imaging throughput is lower in interleaved EPI; 2) the magnitude and phase signal variations among EPI segments (due to physiological noise, subject motion, and B_0_ drift) are translated to significant in-plane aliasing artifact across the field of view (FOV). Here we report a method that integrates multiple approaches to address the technical limitations of interleaved EPI-based fMRI. Firstly, the multiplexed sensitivity-encoding (MUSE) post-processing algorithm is used to suppress in-plane aliasing artifacts resulting from time-domain signal instabilities during dynamic scans. Secondly, a simultaneous multi-band interleaved EPI pulse sequence, with a controlled aliasing scheme incorporated, is implemented to increase the imaging throughput. Thirdly, the MUSE algorithm is then generalized to accommodate fMRI data obtained with our multi-band interleaved EPI pulse sequence, suppressing both in-plane and through-plane aliasing artifacts. The blood-oxygenation-level-dependent (BOLD) signal detectability and the scan throughput can be significantly improved for interleaved EPI-based fMRI. Our human fMRI data obtained from 3 Tesla systems demonstrate the effectiveness of the developed methods. It is expected that future fMRI studies requiring high spatial-resolvability and fidelity will largely benefit from the reported techniques.

## Introduction

Functional magnetic resonance imaging (fMRI) is a non-invasive imaging tool for detecting brain activity using the blood-oxygen-level-dependent (BOLD) contrast [1], [2]. Because of the high temporal resolution of single-shot echo-planar imaging (EPI), the majority of fMRI studies were performed with single-shot EPI [3]. However, it is well known that the spatial resolution and fidelity are limited in single-shot EPI-based fMRI data. Specifically, the geometric distortions of single-shot EPI data make it difficult to accurately align functional data to anatomic references [4], [5]. In addition, the signal decay within the long acquisition window of single-shot EPI broadens the point-spread function (PSF) [6]. Furthermore, it is not always feasible to achieve the optimal echo time (TE) when choosing a large in-plane matrix size for high-resolution fMRI studies [6].

Several approaches may be used to partially address the above-mentioned limitations of single-shot EPI-based fMRI. First, partial-Fourier single-shot EPI may be used to improve the PSF and shorten the minimal TE achievable for high-resolution fMRI [6]. However, the echo-shifting induced signal loss in partial-Fourier gradient-echo EPI may be pronounced in brain regions affected by strong susceptibility field gradients [7], and the geometric distortions remain significant in partial-Fourier single-shot EPI. Second, using parallel imaging techniques, such as the SENSE method [8], EPI distortions can be reduced and the PSF can be improved. Furthermore, the minimal TE achievable can be shortened by parallel EPI, as compared with single-shot full-Fourier EPI. However, the noise may be undesirably amplified by parallel image reconstruction, particularly when choosing a higher acceleration factor (e.g., [4]), resulting in reduction of the BOLD signal detectability. Third, similar to parallel EPI, multi-shot interleaved EPI is superior to single-shot full-Fourier EPI in terms of the reduced geometric distortions, sharper PSF and higher flexibility in achieving the optimal TE. In contrast to parallel EPI, the multi-shot interleaved EPI is not affected by undesirable noise amplification. However, there are two major limitations in interleaved EPI based fMRI: 1) the imaging throughput is lower in interleaved EPI; 2) the magnitude and phase signal variations among EPI segments (due to physiological noise, subject motion, and B_0_ drift) are translated to significant in-plane aliasing artifact across the FOV. Since the aliasing artifact is unstable across dynamic time points, this artifact makes it difficult to reliably detect the BOLD contrast. Although it is possible to use a navigator echo to measure the signal variations among different segments of interleaved EPI [9], the inclusion of navigator echoes may further reduce the imaging throughput. It has been shown recently that parallel imaging, interleaved EPI, and a keyhole scheme can be combined in a flexible way to simultaneously improve the imaging PSF and BOLD signal detectability [10]. In addition, the UNaliasing by Fourier-encoding the Overlaps using the temporal Dimension (UNFOLD) technique can be applied to reduce the aliasing artifact in undersampled, high-throughput EPI data [11]. However, the limitations associated with each of the integrated schemes (e.g., noise amplification associated with parallel imaging) are not eliminated with the reported method [10], and a potential concern with the UNFOLD-based fMRI reconstruction is that a transient head position change at a single imaging time point may be translated to artifacts in multiple imaging time points through temporal-domain data filtering.

Here we integrate multiple approaches to address the technical limitations of interleaved EPI-based fMRI. First, we use the recently developed multiplexed sensitivity-encoding (MUSE) post-processing algorithm, originally designed to remove aliasing artifacts in diffusion-weighted imaging (DWI) [12], to suppress fMRI aliasing artifacts resulting from time-domain signal instabilities. Since the MUSE method does not require any navigator echo, the shot-to-shot signal variations in interleaved EPI-based fMRI can be quantified and corrected without compromising the imaging throughput. Second, we implement a simultaneous multi-band interleaved EPI pulse sequence, with a controlled aliasing scheme incorporated [13], to increase the imaging throughput of interleaved EPI-based fMRI. Third, we further generalize the MUSE reconstruction framework to enable artifact-free and high-throughput fMRI based on our multi-band interleaved EPI pulse sequence.

## Materials and Methods

Our research has been approved by the Institutional Review Board (IRB) at Duke University, and written informed consent has been obtained from the participants. The IRB number is Pro00024624 and the IRB study title is “High resolution intrinsic connectivity network mapping of amygdala functional subdivisions”.

### Removal of in-plane aliasing artifact with multiplexed sensitivity encoding (MUSE)

An implicit assumption in interleaved EPI acquisition and reconstruction is that both the magnitude and phase components of the proton-density source signals are consistent across multiple EPI segments. When this assumption is violated, the signal inconsistencies among EPI segments result in in-plane aliasing artifacts in images reconstructed with 2D Fourier transform. In a recent paper we demonstrated that the impact of shot-to-shot phase inconsistencies on interleaved EPI-based DWI can be minimized with a MUSE algorithm [12], which can be briefly summarized as a 3-step procedure. First, aliasing-free images are reconstructed from each of the EPI segments using the conventional SENSE algorithm [8]. Although the noise is undesirably amplified by the SENSE method, the reconstructed images still provide reliable information on the phase variations, which are slowly varying in space, among multiple EPI segments. Second, the phase information obtained from step 1 is spatially smoothed and thus de-noised. Third, the smoothed phase information obtained from step 2 (i.e., 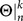 in Equation 1) and the known coil sensitivity profiles (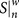) are incorporated into a mathematical framework, as shown in Equation 1, that jointly solves the unknown magnitude source signals (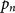) of overlapping voxels from all EPI segments (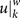).
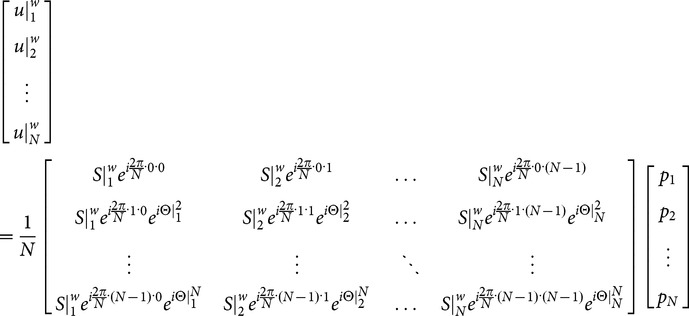
(1)where 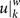 represents the aliased image-domain signal (i.e., from a certain voxel in reduced-FOV image) of the *k*-th EPI segment (k = 1,2,…N; with N being the total number of EPI segments) measured with coil number *w* (*w* = 1,2,…*W*; with *W* being the total coil number); 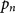 represents unaliased full-FOV image signal at location *n* (*n* = 1…*N*; separated by 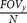 along the phase-encoding direction); 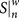 represents the known coil sensitivity profile for coil number *w* (*w* = 1,2,…*W*; with *W* being the total coil number) at location *n*; 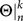 represents the shot-to-shot phase inconsistency (e.g., due to motion or B_0_-drift) at location *n* for the *k*-th EPI segment.

Note that Equation 1 is the generalized form of the MUSE reconstruction for N-shot interleaved EPI, and Equations 9 and 10 shown in [12] are for 2-shot interleaved EPI that can be obtained by setting N = 2 in Equation 1 above. The matrix inversion conditioning in step 3 is greatly improved as compared with the conventional SENSE method, thus avoiding undesirable noise amplification associated with the original SENSE reconstruction. For example, for 4-shot interleaved EPI data acquired with an 8-channel RF coil, the MUSE reconstruction solves 4 unknowns with 32 equations (instead of solving 4 unknowns with 8 equations using SENSE).

The application of MUSE to remove aliasing artifact resulting from phase inconsistencies among EPI segments has been previously demonstrated for DWI [12]. We expect that the MUSE method should also be capable of removing the in-plane aliasing artifacts due to inter-segment phase variations in interleaved EPI-based fMRI (and other types of dynamic scans), and thus can improve the time-domain BOLD signal detectability. Furthermore, although MUSE still implicitly assumes that the magnitude signals are uniform across multiple EPI segments, we hypothesize that the algorithm, with its inclusion of coil sensitivity profiles in the constrained reconstruction, is capable of suppressing in-plane aliasing artifacts resulting from inter-segment magnitude signal variations (e.g., due to physiological noise or motion) as compared with the conventional interleaved EPI reconstruction. In this paper we conduct numerical simulations and human studies to assess if the MUSE reconstruction can improve interleaved EPI-based fMRI, in the presence of magnitude, phase and position variations across EPI segments (see the sections on “*numerical simulations of single-band MUSE reconstruction in the presence of signal instabilities*” and “*Improvement of EPI time-domain signal stability by single-band MUSE*”).

### Simultaneous multi-band imaging for interleaved EPI

Here we incorporate simultaneous multi-band imaging [14], [15] into an interleaved EPI pulse sequence, so that the TR can be shortened for a fixed number of slices and thus the imaging throughput can be improved for interleaved EPI-based fMRI scans.

It has been shown that if the FOVs corresponding to the simultaneously excited slices are shifted differently, then the parallel MRI reconstruction of simultaneous multi-band imaging is less susceptible to noise amplification associated with parallel reconstruction for two reasons. First, the number of overlapping voxels from multiple slices is smaller with FOV shifting; Second, the coil sensitivity profiles for the overlapping voxels of FOV-shifted images are less similar, resulting in improved matrix inversion conditioning [13]. For example, incorporation of the CAIPIRINHA method can improve the reliability of multi-band single-shot fMRI reconstruction [16].

**Figure 1:**
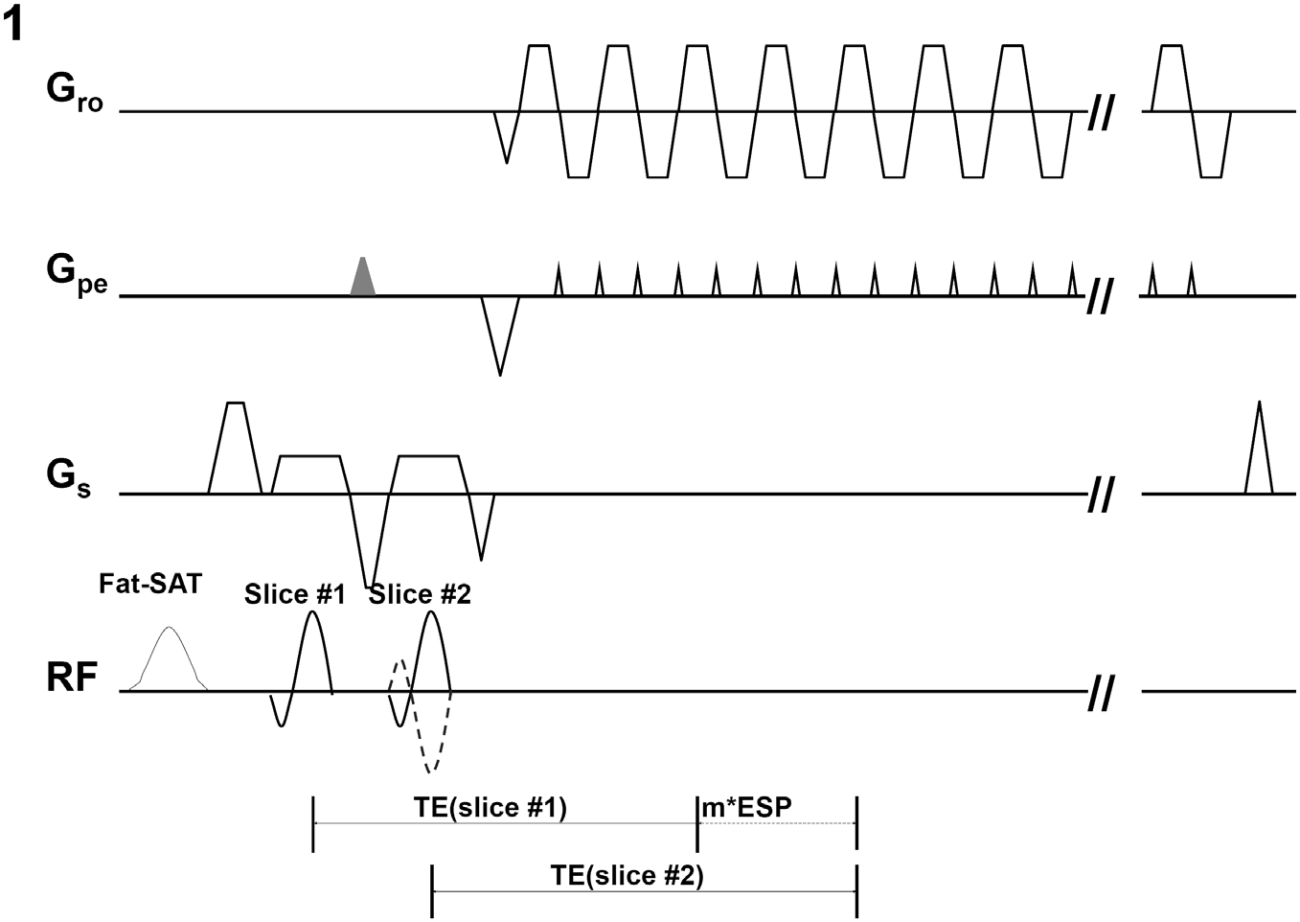
The simultaneous 2-band interleaved EPI pulse sequence with TE compensation of first slice, where m is an integer and ESP is the echo-spacing time of EPI echo train. The TE compensation of the first slice is the multiples of ESP.

Our implemented multi-band 2-shot interleaved EPI pulse sequence, with a controlled aliasing scheme incorporated, is shown in Fig. 1. In this gradient echo EPI sequence, a pair of slices is excited within the same TR by two consecutive RF excitation pulses (placed after a spectrally-selective fat saturation RF pulse). An additional pre-phasing gradient along the phase-encoding direction (i.e., the gray trapezoid in Fig. 1) is added between two RF pulses to ensure that comparable echo times can be achieved for the two excited slices. For 2-shot interleaved acquisition, the polarity of the second excitation RF pulse alternates between two consecutive TRs (i.e., solid and dashed lines in Fig. 1), shifting the FOV by half for one of the simultaneously excited slices. For interleaved EPI with a larger number of shots (e.g., 4), we can further modulate the phase terms across multiple shots to shift the FOV differently for the excited slices. In this paper we implement and evaluate the multi-band interleaved EPI in fMRI studies.

### Generalization of MUSE algorithm to multi-band interleaved EPI

The MUSE algorithm originally designed for non-multi-band imaging (i.e., Equation 1) can be generalized to accommodate interleaved EPI data acquired with simultaneous multi-band imaging. Similar to the original MUSE method, the generalized MUSE algorithm comprises three steps. First, images free from both in-plane and through-plane aliasing artifacts are reconstructed from each of the multi-band EPI segments using the SENSE algorithm. Second, the phase information obtained from step 1 is spatially smoothed and thus denoised. Third, the smoothed phase information (from each of the simultaneously excited slices) obtained from step 2 and the known coil sensitivity profiles are incorporated into a mathematical framework, as shown in Equation 2, that jointly solves the unknown magnitude source signals of (in-plane and through-plane) overlapping voxels from all EPI segments (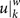). To simplify the illustration, in Equation 2 we use 2-band imaging as an example.
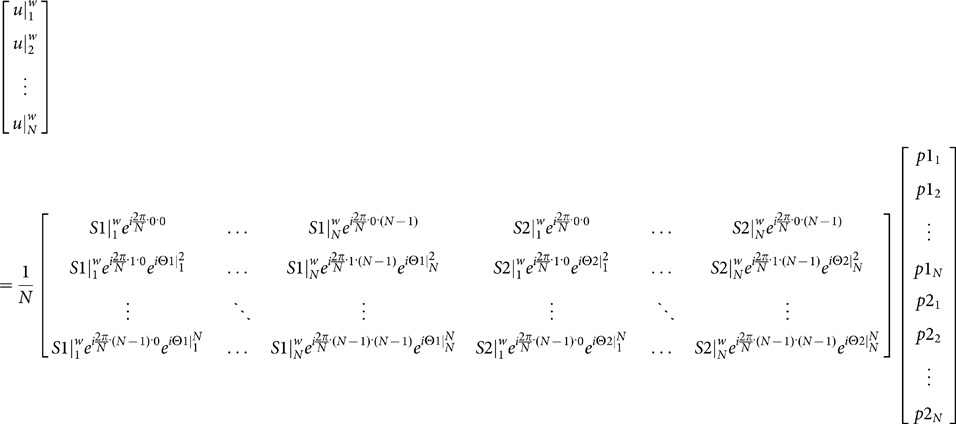
(2)where 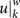 represents the aliased and multi-band image-domain signal (i.e., with both in-plane and through-plane aliasing artifacts) of the *k*-th EPI segment (k = 1,2,…N; with N being the total number of EPI segments) measured with coil number *w* (*w* = 1,2,…*W*; with *W* being the total coil number); 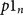 represents unaliased full-FOV image signal at location *n* (*n* = 1…*N*; separated by 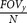 along the phase-encoding direction) from the first slice of the simultaneously excited slices; 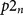 represents unaliased full-FOV image signal at location *n* (*n* = 1…*N*; separated by 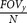 along the phase-encoding direction) from the second slice of the simultaneously excited slices; 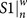 and 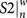 represent the known coil sensitivity profiles for coil number *w* (*w* = 1,2,…*W*; with *W* being the total coil number) at location *n* from the first slice and the second slice, respectively, of the simultaneously excited slices; 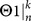 and 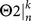 represent the phase errors due to motion or B_0_-drift at location *n* for the *k*-th EPI segment from the first slice and the second slice, respectively, of the simultaneously excited slices. Note that the multi-band MUSE algorithm shown in Equation 2 can be easily extended to accommodate multi-band interleaved EPI data with a larger number of simultaneously excited slices. In this paper we demonstrate and evaluate the multi-band MUSE reconstruction algorithm in fMRI studies. The MUSE-based interleaved fMRI reconstruction was evaluated in a series of numerical simulations and human experiments, with data obtained from 10 healthy adult volunteers using our 3 Tesla MRI scanner (GE, Waukesha, WI, USA).

### The PSF of images reconstructed with SENSE and MUSE

The multi-shot EPI has a sharper PSF as compared with single-shot EPI, and thus can provide images with higher resolution and resolvability. Here we use a numerical simulation to investigate if the PSF of SENSE- and MUSE-produced multi-shot EPI images remains sharp.

In our simulation the signals decay exponentially (with T2* value = 40 msec) during EPI k-space data acquisition. Firstly, the theoretical PSF waveforms of full-Fourier EPI k-space data with 128 phase encoding steps were calculated for three acquisition schemes with different inter ky-line echo-spacing time (ESP): 1) single-shot EPI (with ESP = 1.2 msec), 2) 2-shot interleaved EPI (with ESP = 0.6 msec), and 3) 4-shot interleaved EPI (with ESP = 0.3 msec). The full-width-half-maximum (FWHM) values of the PSF waveforms were quantified. Secondly, RF coil sensitivity profiles of eight coil elements were simulated and used to create multichannel MRI data. The PSF waveforms in images reconstructed with either SENSE or MUSE were calculated, and the FWHM values were measured by fitting a Lorentz function to the PSF waveforms. Thirdly, the FWHM of 1) the theoretical PSF waveforms, 2) the PSF waveforms in SENSE produced images, and 3) the PSF waveforms in MUSE produced images were compared.

### Numerical simulations of single-band MUSE reconstruction in the presence of signal instabilities

To assess the single-band MUSE performance in the presence of time domain signal instabilities resulting from various sources (e.g., magnitude, phase, and position variations), we systematically conducted a series of numerical simulations by mathematically adding inter-segment signal variations to a 4-shot interleaved EPI k-space data set (i.e., the gold standard of our simulation), which was obtained from a healthy volunteer using an 8-channel coil with minimal intrascan motion and no in-plane aliasing artifact, as follows:

1. Phase signal variation: Different image-domain constant phase terms were added to four identical copies of 4-shot interleaved EPI data. Then, four sets of k-space data were calculated from the images (through inverse 2D Fourier transform), and a single composite 4-shot EPI k-space data set was produced by combining subsets of k-space data from four different copies (i.e., mathematically combining the first EPI segment from the first copy, the second EPI segment from the second copy, the third EPI segment from the third copy, and the fourth EPI segment from the fourth copy). We systematically changed the levels of inter-segment phase variations (ranging from 0.015 to 0.3 radians in 20 steps) in our simulation.
2. Magnitude signal variation: A mask comprising mostly voxels of gray matter (GM) and cerebrospinal fluid (CSF) was first generated from our gold standard image by applying a signal-intensity threshold. Using a procedure similar to the phase variation simulation, we changed the image-domain magnitude values of voxels within the GM-CSF mask in each EPI segment, producing composite 4-shot EPI data affected by inter-segment magnitude variations. We then repeated this simulation with different levels of inter-segment magnitude variations, ranging from 2% to 40% (in 20 steps) of the original signal intensity of each voxel.
3. Position change: Using a simulation procedure similar to the one described above, different image-domain position changes were applied to four identical copies of 4-shot interleaved EPI data. Specifically, clockwise and counterclockwise rotations were added to the first two and the last two copies of interleaved EPI data, respectively. The translational motions along the frequency encoding direction were then added to the rotated data. Afterward, a composite 4-shot EPI k-space data set was produced by combining k-space subsets of the four data sets. The simulated 4-shot EPI data sets were affected by inter-segment rigid-body position changes comprising a fixed level of translational motion (1 pixel along the frequency encoding direction) and various levels of rotational motion (0.25 to 5 degrees in 20 steps).
4. Time-domain signal variations from multiple sources: We created simulated 4-shot EPI data sets affected by inter-segment signal variations resulting simultaneously from multiple sources: magnitude variation, phase variation, and position change.

The simulated k-space data sets with different levels of inter-segment signal variations were reconstructed with two different approaches: 1) 2D Fourier transform (i.e., conventional interleaved EPI reconstruction) and 2) the MUSE algorithm. The reconstructed images were then compared with the gold standard magnitude image, and the artifact levels (i.e., the L1-norm of the difference between reconstructed images and the gold standard) were measured.

### Improvement of EPI time-domain signal stability by single-band MUSE

In this section we experimentally demonstrate that the single-band MUSE algorithm can effectively remove in-plane aliasing artifacts and improve time-domain signal stability in single-band multi-shot fMRI data obtained from an 8-channel RF phase-array head coil with its coil geometry supporting 1D parallel imaging. The single-band MUSE method is the foundation for multi-band MUSE, which is applicable only to multi-band multi-shot fMRI data obtained with RF coils that support 2D parallel imaging (see the section on “Multi-band interleaved EPI based fMRI”).

4-shot interleaved EPI data were acquired from nine healthy volunteers using an 8-channel RF phase-array head coil. Imaging parameters included: in-plane matrix size 140×140, FOV 21×21 cm^2^, thickness 3 mm, TR 2 sec, and TE 25 msec. The scan duration was 5 min (i.e., 150 RF pulse excitations) for each run. After Nyquist artifact correction based on the phase-cycled reconstruction algorithm [17], the acquired 4-shot interleaved EPI data underwent three different reconstruction procedures, as described below, producing multiple sets of images to be quantitatively assessed.

The first is conventional interleaved EPI reconstruction: k-space data obtained from every four consecutive RF pulse excitations were assembled and Fourier transformed (i.e., sliding window of 4 consecutive EPI segments: [1], [2], [3], [4], [2], [3], [4], [5]…[147,148,149,150]), producing 147 sets of full-FOV images.

The second is SENSE reconstruction: By applying the SENSE algorithm to 4× subsampled k-space data corresponding to each RF pulse excitation, 150 sets of full-FOV images were reconstructed from the acquired 4-shot EPI data. These 150 images were then temporally smoothed, with a sliding window of 4 time points, so that the final images have the same smoothing effect as that in images produced from the conventional interleaved EPI reconstruction described in the previous paragraph.

The third is MUSE reconstruction: Similar to the conventional interleaved EPI reconstruction procedure, 4× subsampled k-space data obtained from every four consecutive RF pulse excitations of interleaved EPI scan were assembled ([1], [2], [3], [4], [2], [3], [4], [5]…[147,148,149,150]). These assembled full-Fourier k-space data then underwent the MUSE reconstruction, producing 147 sets of full-FOV images without aliasing artifacts.

The temporal fluctuation noise level (i.e., the time-domain standard deviation) and the signal-to-fluctuation noise ratio (SFNR), both indicative of fMRI data quality, were then calculated from the last 145 time points of dynamic images produced from three different reconstruction procedures.

In this section, sliding window averaging was used, so that images reconstructed with different algorithms with inherently different temporal resolutions can be objectively compared. It should be noted that the developed MUSE algorithm is applicable to multi-shot fMRI reconstruction either with or without sliding window averaging (see the next section on “Hybrid simulation studies of fMRI time-domain signal fidelity”).

### Hybrid simulation studies of fMRI time-domain signal fidelity

A hybrid simulation study was conducted to quantitatively assess the impact of multi-band MUSE reconstruction on fMRI time-domain signal fidelity. Finger-tapping full k-space fMRI data obtained with single-shot EPI using a 32-channel phase array coil were used as the gold standard. The scan duration was 5 min, comprising alternating resting blocks (30 sec each) and finger-tapping blocks (30 sec each). Imaging parameters included: in-plane matrix size 96×96, FOV 21×21 cm^2^, thickness 3.0 mm, TR 2.0 sec, and TE 35 msec. k-Space signals of four axial slices from the gold standard data set were used to simulate three sets of sub-sampled k-space data: 1) single-band 2-shot EPI, 2) 2-band 2-shot EPI, and 3) 2-band 4-shot EPI. Images reconstructed from the hybrid simulation k-space data, using either MUSE or the SENSE algorithm, were then compared with the gold standard data set. The sliding-window averaging was not applied to this hybrid simulation to avoid the change of time-domain noise correlation patterns in reconstructed images. The time-domain signal fidelity in MUSE and SENSE produced images were measured by 1) the voxel-wise correlation coefficients between the reconstructed time course profiles and the gold standard time course profiles in activated voxels, and 2) the normalized L1-norm of the difference between reconstructed images and the gold standard.

### Multi-band interleaved EPI-based fMRI

In order to effectively remove both in-plane and through-plane aliasing artifacts in multi-band multi-shot fMRI data with the developed multi-band MUSE algorithm, we needed to use RF coils that support 2D parallel imaging, such as the Nova 32-channel coil (8 by 4). With the 8 by 4 coil geometry of our 32-channel coil, we were able to produce high-quality axial images from 4-shot 2-band fMRI scans using the multi-band MUSE reconstruction, as confirmed by our hybrid simulation shown in the previous section. In this section we choose to use 2-shot 2-band fMRI protocol, in order to achieve the desired spatial-temporal-resolution in experimental fMRI studies.

To evaluate the generalized multi-band MUSE algorithm in fMRI studies, two sets of finger tapping fMRI data (of both hands) with different spatial resolutions (2×2×3 mm^3^, and 1.8×1.8×1.8 mm^3^) were acquired from two healthy volunteers using a 2-band and 2-shot interleaved EPI pulse sequence (as shown in Fig. 1) with a Nova 32-channel phase array RF coil. The scans were 5 min and 8 min in duration, and consisted of alternating resting blocks (30 sec each) and finger-tapping blocks (30 sec each). Imaging parameters included: in-plane matrix size 128×128, FOV 25.6×25.6 cm^2^ and 23.1×23.1 cm^2^, thickness 3 mm and 1.8 mm, TR 2 sec and 1.5 sec, and inter-*k_y_* line echo spacing time 0.62 msec and 0.65 msec. The two simultaneously excited slices have comparable TE values: 24.86 and 25 msec (for 2×2×3 mm^3^), 29.26 and 30 msec (for 1.8×1.8×1.8 mm^3^), using the pulse sequence design shown in Fig. 1. With this 2-band and 2-shot interleaved EPI scheme, 128×64 *k_y_* lines from a pair of simultaneously excited slices were acquired in each shot, and 22 shots (covering 44 axial slices with 2 sec TR) or 20 shots (covering 40 axial slices with 1.5 sec TR) were performed in each TR. k-Space data from two consecutive TRs were assembled, and then underwent either 1) the conventional reconstruction (i.e., 2D Fourier transform followed by unfolding the through-plane signals with SENSE) or 2) the multi-band MUSE reconstruction algorithm (Equation 2). Furthermore, a multi-band UNFOLD technique was applied to the high-resolution fMRI data set (1.8×1.8×1.8 mm^3^ voxel size) with the following procedures: 1) Removal of EPI Nyquist ghost artifacts, 2) UNFOLD based temporal-domain filtering for removing aliasing artifacts along the phase-encoding direction, and 3) SENSE-based reconstruction along the slice-select direction for removing through-plane aliasing artifacts. The SFNR values were then calculated from the multi-band UNFOLD produced images.

Preprocessing of fMRI data was conducted using tools from the Oxford Centre for Functional MRI of the Brain’s Software Library (FSL version 5.0.1, www.fmrib.ox.ac.uk/fsl) and locally developed Matlab code (MathWorks, Natick, MA). The first three volumes were discarded in order to allow for stabilization of MRI signals. The data were slice-time corrected and then realigned to correct for head movements. fMRI activation maps were calculated using the FSL-FEAT program without spatial smoothing.

To compare fMRI results obtained from interleaved EPI and conventional single-shot EPI protocols, a single-shot EPI-based fMRI dataset (finger tapping of both hands) with 3.75×3.75×3.8 mm^3^ voxel size was acquired from one of the recruited healthy volunteers. The scan duration was 5 min, consisted of alternating resting blocks (30 sec each) and finger-tapping blocks (30 sec each). Imaging parameters included: in-plane matrix size 64×64, FOV 24.0×24.0 cm^2^, thickness 3.8 mm, TR 1.5 sec, and TE 30 msec. The fMRI activation maps were calculated using the FSL-FEAT program (without spatial smoothing).

## Results

### The PSF of images reconstructed with SENSE and MUSE

**Figure 2:**
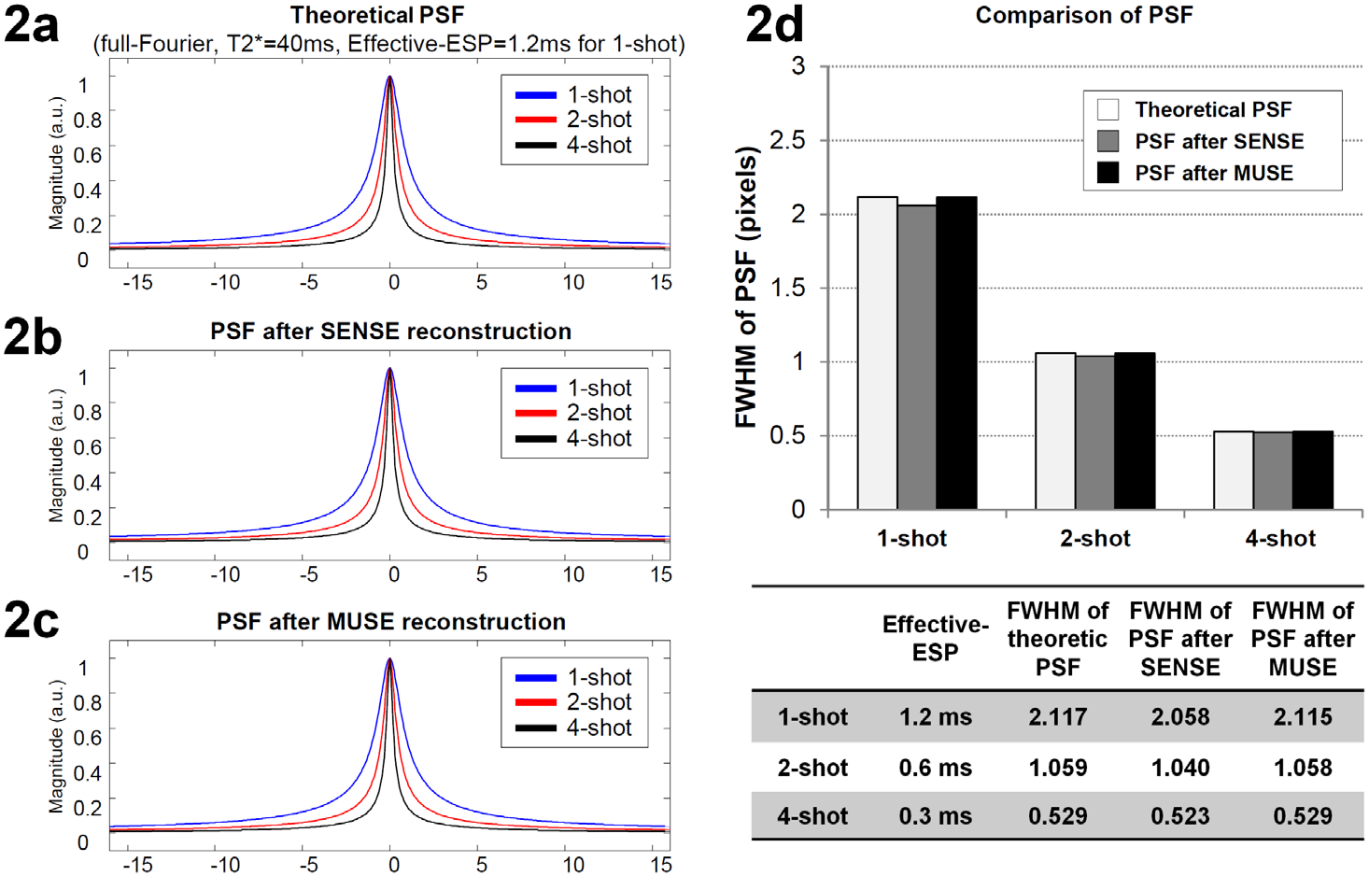
Mathematical simulation of the point spread function (PSF) for MUSE reconstruction: (a) The theoretic PSF, (b) the PSF of SENSE-produced images, and (c) the PSF of MUSE-produced images corresponding to different numbers of EPI segments. (d) The FWHM values of PSF waveforms corresponding to different acquisition and reconstruction schemes.

Fig. 2a to 2c show the theoretical PSF waveforms, PSF of SENSE-produced images, and PSF of MUSE-images, respectively, corresponding to different numbers of EPI segments. Fig. 2d quantitatively compares the FWHM values of PSF waveforms corresponding to different acquisition and reconstruction schemes. It can be seen that the PSF remains sharp in MUSE-produced images, as compared with the theoretical PSF, for different interleaved EPI acquisition schemes.

### Numerical simulations of single-band MUSE reconstruction in the presence of signal instabilities

**Figure 3:**
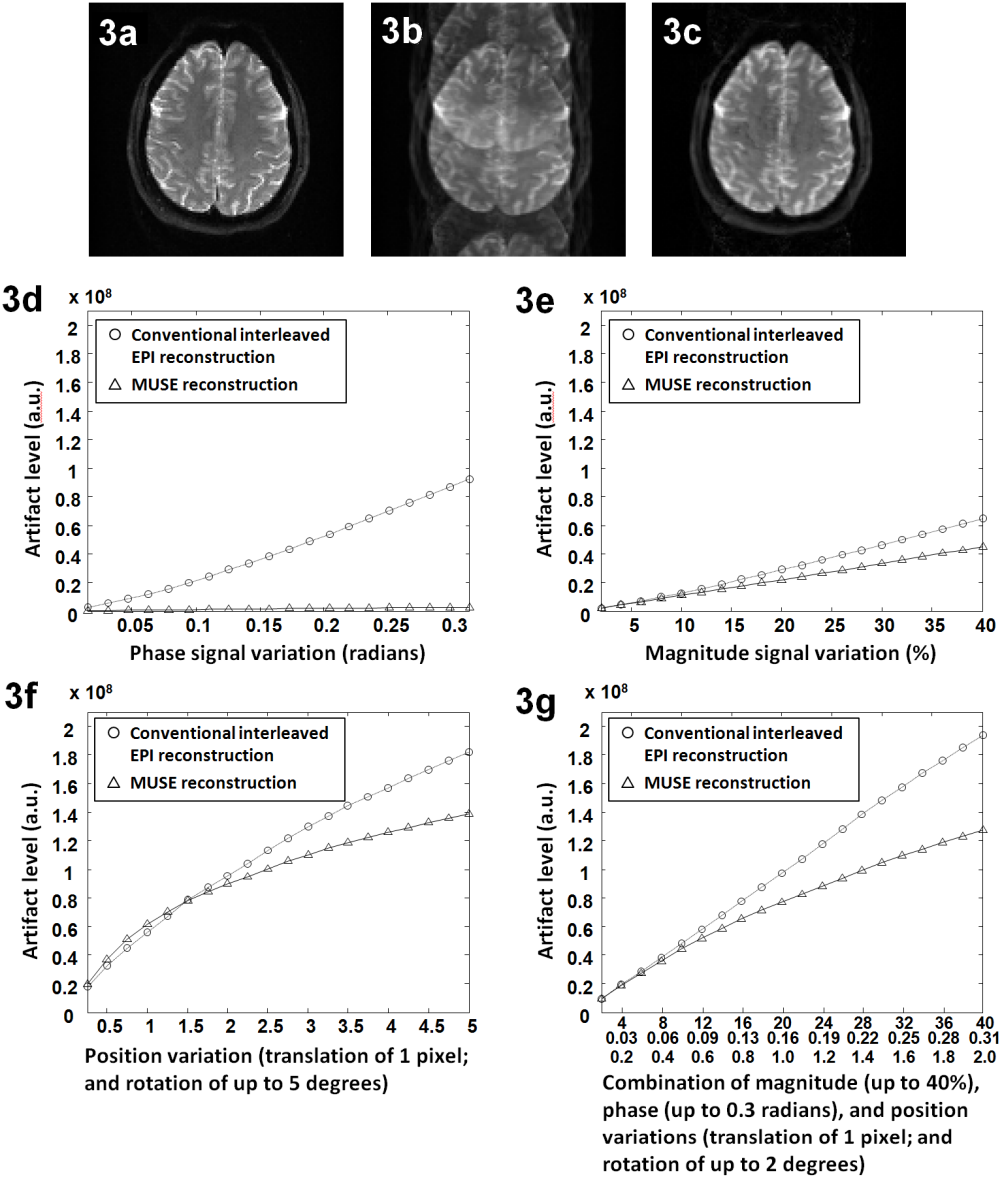
The impact of data inconsistencies on MUSE produced image quality: (a) The gold standard of our numerical simulation. (b) The conventional interleaved EPI in the presence of inter-segment phase variation (0.3 radians), magnitude variation (20%), translational motion (0.5 pixel) and rotational motion (1 degree). (c) The MUSE reconstruction of the same data set shown in b. (d) Artifact levels in images reconstructed with Fourier transform (i.e., conventional interleaved EPI: circles) and MUSE (triangles) in the presence of inter-segment phase variations. (e) Artifact levels in images reconstructed with Fourier transform (circles) and MUSE (triangles) in the presence of inter-segment magnitude variations. (f) Artifact levels in images reconstructed with Fourier transform (circles) and MUSE (triangles) in the presence of inter-segment position changes (at a fixed level of translation of 1 pixel and various levels of rotation). (g) Artifact levels in images reconstructed with Fourier transform (circles) and MUSE (triangles) in the presence of inter-segment signal variations simultaneously resulting from multiple sources.

Fig. 3a shows the gold standard of our numerical simulation, and Fig. 3b shows a simulated 4-shot interleaved EPI image (reconstructed with a simple 2D Fourier transform) in the presence of the combination of inter-segment phase variation (1.05 radians), magnitude variation (20% in GM and CSF areas), translational motion (0.5 pixel) and rotational motion (1 degree). Significant in-plane aliasing artifacts resulting from inter-segment signal variations in this interleaved EPI data set can be seen. As shown in Fig. 3c, the aliasing artifact can be greatly reduced (by 77%) in the image reconstructed from the same k-space data set shown in b using the MUSE algorithm.

Fig. 3d to 3g show the artifact levels in images reconstructed with Fourier transform (circles) and MUSE (triangles) corresponding to different levels of phase variations (d), magnitude variations (e), position changes comprising a fixed level of translation and various levels of rotations (f), and the combinations of all three sources (g). It can be seen that, in most cases, the residual artifact is lower in images reconstructed with the MUSE algorithm in comparison to the conventional interleaved EPI reconstruction. As expected, the artifacts resulting from phase inconsistencies can be almost completely eliminated by the MUSE method (d). We also note that the artifacts resulting from inter-segment magnitude and position variations can also be reduced in MUSE reconstructed images (e to g).

### Improvement of EPI time-domain signal stability by single-band MUSE

**Figure 4:**
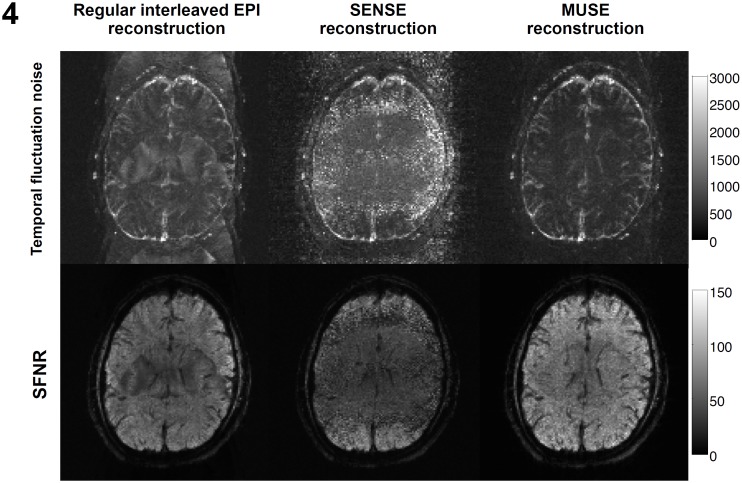
Time-domain signal stabilities, measured by the temporal fluctuation noise (upper row) and SFNR (lower row), of images reconstructed with conventional interleaved EPI reconstruction (left), the SENSE algorithm (middle), and the MUSE algorithm (right).

As shown in the top row of Fig. 4, the conventional interleaved EPI reconstruction is highly susceptible to unstable aliasing artifacts, as reflected by the aliasing patterns in the temporal fluctuation noise map (left: 863±254). Using the SENSE reconstruction (middle: 1219±490), the temporal noise is high overall since the parallel image reconstruction is susceptible to undesirable noise amplification when choosing a higher acceleration factor (e.g., 4). As compared with the conventional interleaved EPI and SENSE reconstruction, the MUSE algorithm is capable of producing images with significantly lower temporal fluctuation noise (right: 680±263).

The SFNR maps obtained with three different reconstruction procedures are shown in the bottom row of Fig. 4. It can be seen that the MUSE reconstruction can produce dynamic images with higher SFNR (right: 61±18), as compared with the interleaved EPI (left: 47±14) and SENSE (middle: 35±14) reconstruction of the same data set. The quantitative comparison of three different reconstruction procedures (Fig. 4a: using data from one of the participants) indicates that the best fMRI quality and BOLD signal detectability can be achieved with the MUSE reconstruction of interleaved EPI data. Similar results were obtained from data of all 9 participants.

### Hybrid simulation studies of fMRI time-domain signal fidelity

**Figure 5:**
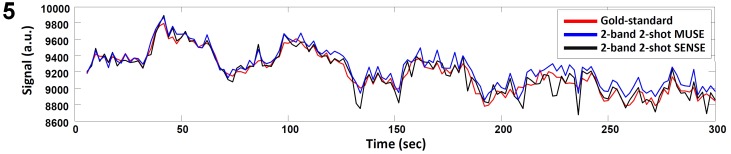
Time course profiles of an activated voxel in the gold standard data set, 2-band 2-shot MUSE reconstructed images, and 2-band 2-shot SENSE reconstructed images.

Fig. 5 shows the time course profiles from an activated voxel of the gold standard data set (red), 2-band 2-shot MUSE produced images (blue), and 2-band 2-shot SENSE produced images (black). The correlation coefficients between the gold standard time course profile and the time course profiles obtained with different reconstruction methods are 0.9997 (for single-band 2-shot MUSE), 0.9623 (for 2-band 2-shot MUSE), 0.9588 (for 2-band 4-shot MUSE), and 0.9387 (for 2-band 2-shot SENSE). These results demonstrate that the MUSE reconstruction can produce fMRI data with higher time-domain signal fidelity than the SENSE reconstruction.

The normalized L1 norm of the difference between the gold standard and other reconstruction methods are 0.25% (for single-band 2-shot MUSE), 0.87% (for 2-band 2-shot MUSE), 0.98% (for 2-band 4-shot MUSE), and 1.12% (for 2-band 2-shot SENSE). These data further illustrate that the MUSE-produced time-domain data have higher fidelity for both activated and non-activated voxels as compared with the SENSE produced images.

### Multi-band interleaved EPI-based fMRI

**Figure 6:**
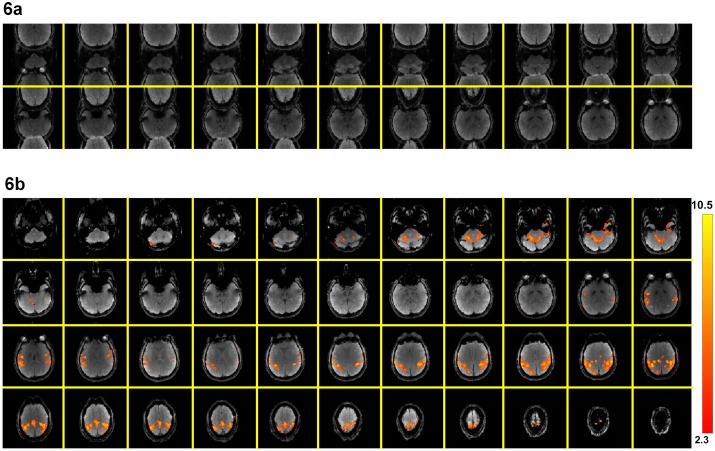
Comparison of images before and after multi-band MUSE reconstruction: (a) Multi-band images reconstructed by directly applying 2D Fourier transform to a full k-space data set (obtained from two consecutive TRs). (b) Images obtained with the multi-band MUSE reconstruction algorithm, showing 44 axial slices without in-plane and through-plane aliasing artifacts. Functional activation is displayed by color.

Multi-band images reconstructed by directly applying 2D Fourier transform to a full k-space data sets (i.e., by assembling the k-space data from two consecutive TRs) with 2.0×2.0×3.0 mm^3^ voxel size are shown in Fig. 6a, where signals from two distant axial slices overlapped, as expected. It can be seen that signals from one of the simultaneously excited slices are shifted by half of the FOV, because the RF pulse polarity changes between 2 EPI segments for one of the slices (Fig. 1). Images obtained with the multi-band MUSE reconstruction algorithm are shown in Fig. 6b, where signals from all 44 axial slices are correctly reconstructed without in-plane and through-plane aliasing artifacts. Functional activation associated with the finger-tapping task can be properly identified from dynamic images produced with the multi-band MUSE algorithm, as shown by color voxels (overlaid on one of the multi-band MUSE produced images in Fig. 6b).

**Figure 7:**
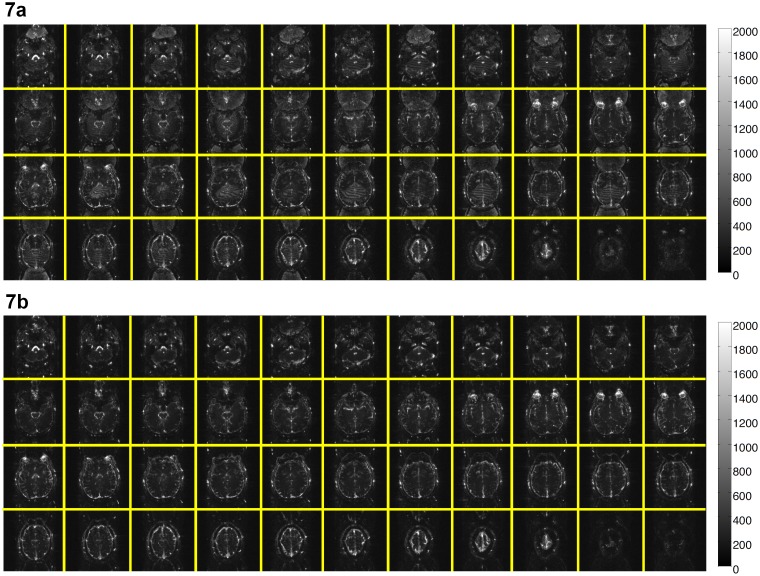
Comparison of time domain signal stability with conventional and multi-band MUSE reconstruction: (a) The temporal fluctuation noise levels in multi-band interleaved EPI data reconstructed with the conventional procedure. (b) The temporal fluctuation noise levels in multi-band interleaved EPI data reconstructed with the MUSE algorithm.

**Figure 8:**
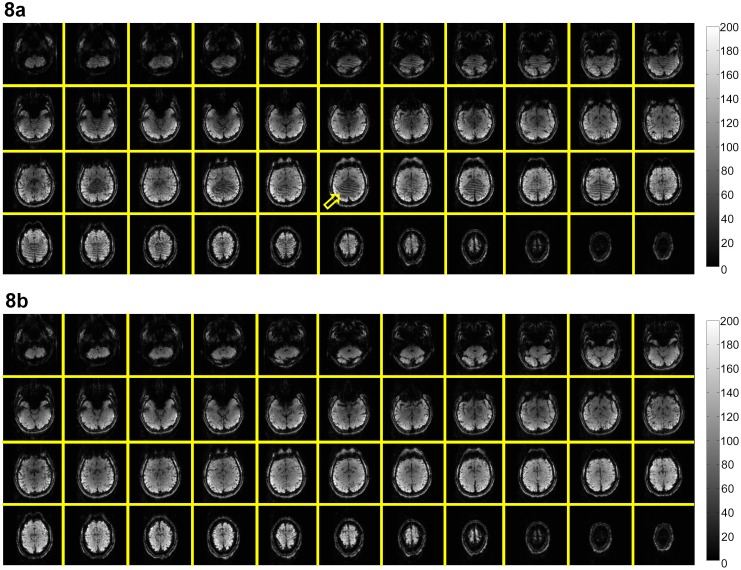
Comparison of SFNR with conventional and multi-band MUSE reconstruction: (a) The SFNR values of multi-band interleaved EPI data reconstructed with the conventional procedure. (b) The SFNR values of multi-band interleaved EPI data reconstructed with the MUSE algorithm.

Although the conventional reconstruction procedure (i.e., 2D Fourier transform followed by separating the simultaneously excited slices with SENSE) can produce images without through-plane aliasing artifact, quantitative measurements show that the MUSE reconstruction can produce images with lower in-plane aliasing artifact and lower time-domain signal fluctuation (by 12.5% in our 2-band and 2-shot EPI data acquired with a 32-channel coil). Fig. 7a and 7b compare the temporal fluctuation noise levels in images reconstructed with the conventional procedure (246±29) and the MUSE algorithm (215±20), respectively. Fig. 8a and 8b show the SFNR measures corresponding to the conventional reconstruction (62±22) and the MUSE reconstruction (69±23), respectively. The results shown in Figs. 6–8 indicate that the quality of high-throughput and high-resolution fMRI, acquired with the multi-band interleaved EPI pulse sequence, can be significantly improved by the developed MUSE algorithm. Artifacts in images obtained with the conventional reconstruction method (e.g., indicated by an arrow in Fig. 8a) can be effectively removed with the MUSE algorithm.

**Figure 9:**
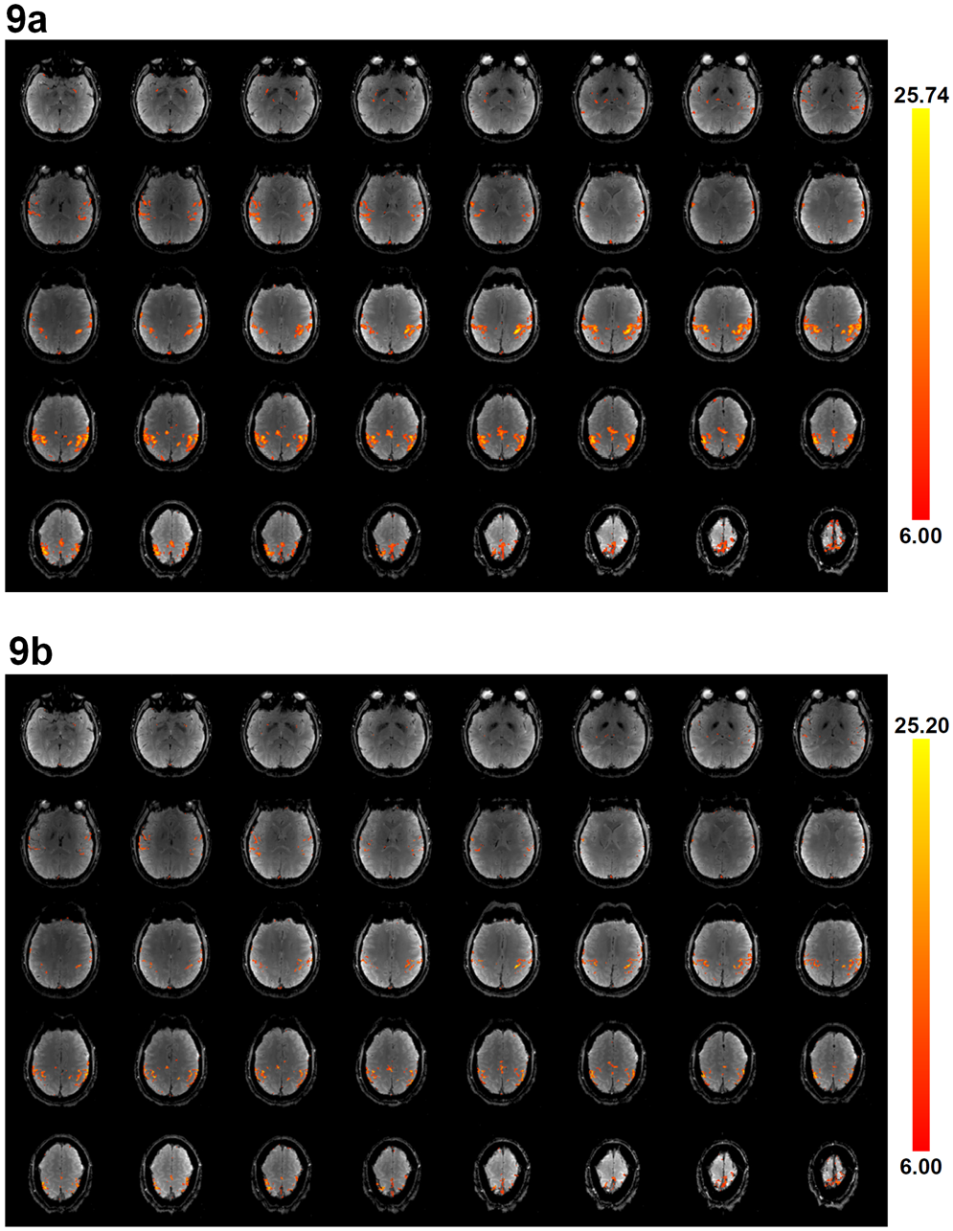
High-resolution multi-band images (1.8×1.8×1.8 mm^3^) reconstructed by (a) the multi-band MUSE algorithm, and (b) the multi-band UNFOLD technique, showing 40 axial slices without in-plane and through-plane aliasing artifacts. Functional activation is displayed on top of MUSE-reconstructed and UNFOLD-reconstructed images.

**Figure 10:**
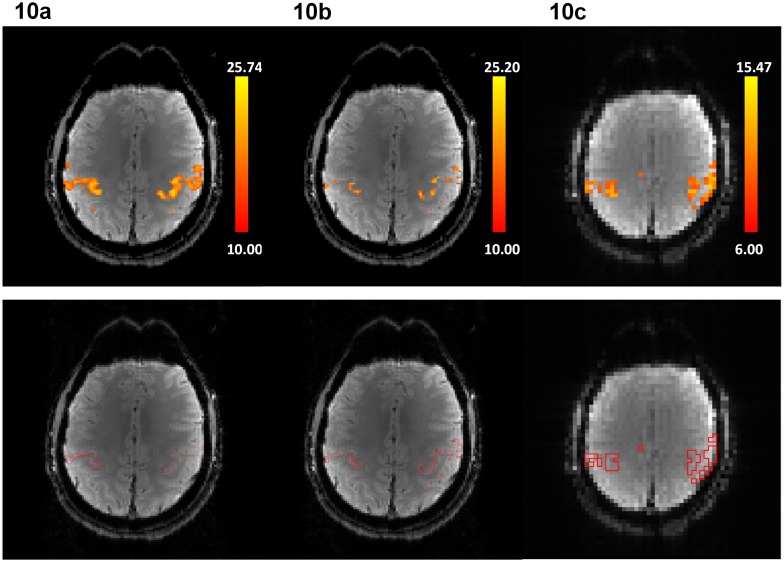
Functional activation maps derived from (a) 2-band 2-shot MUSE-fMRI (1.8×1.8×1.8 mm^3^), (b) multi-band UNFOLD fMRI data (1.8×1.8×1.8 mm^3^) and (c) conventional single-shot EPI based fMRI (3.75×3.75×3.8 mm^3^) are shown in the upper row. The contour lines of the activation areas are shown in the lower row.

High-resolution fMRI data (1.8×1.8×1.8 mm^3^) obtained with the multi-band MUSE algorithm and the multi-band UNFOLD technique are shown in Fig. 9a and 9b, respectively, where signals from all 40 axial slices are correctly reconstructed without in-plane or through-plane aliasing artifacts. Functional activation associated with the finger-tapping task can be properly identified from dynamic images produced with either the multi-band MUSE algorithm or the multi-band UNFOLD technique (shown by color voxels), although the functional activation obtained from the multi-band MUSE algorithm has higher statistical power than that reconstructed by the multi-band UNFOLD technique. Fig. 10a, 10b and 10c show multi-band MUSE produced high-resolution fMRI activation, multi-band UNFOLD produced high-resolution fMRI activation, and conventional low-resolution fMRI activation, respectively. Because the scan durations, voxel size and SNR were different between high-resolution and low-resolution fMRI scans, different statistical thresholds were chosen to achieve the same total activation area across two slices (in Fig. 10a and 10c) covering major sensorimotor areas. Based on the contour lines of the activation (shown in the lower row of Fig. 10), it can be seen that MUSE-produced high resolution fMRI data can better differentiate gray and white matters, and are less affected by the partial volume effect as compared with low resolution fMRI data.

## Discussion

Recent studies have demonstrated that, by increasing the spatial-resolvability of fMRI (dependent on the voxel size and PSF), previously unknown function or undefined connectivity networks can be measured. For example: First, brainstem nuclei of the dopamine, norepinephrine, and serotonin systems have long been known to play a critical role in the regulation of brain function, and disturbances of these systems have been implicated in most major psychiatric and neurological disorders. However, using conventional low-resolution fMRI protocols (e.g., 4 mm^3^ isotropic), functional activity in small nuclei cannot be measured. On the other hand, using fMRI of higher spatial-resolution (e.g., 1.5 mm^3^ isotropic), the BOLD responses from individual dopaminergic brainstem nuclei can be studied and its functional distinction from projected brain regions can be characterized in humans [18]. Second, based on high-resolution fMRI data (2 mm^3^ isotropic), Vincent et al. confirmed the existence of a previously unknown frontoparietal control network, which is anatomically positioned to integrate information from two opposing brain systems: the dorsal attention system and the hippocampal-cortical memory system [19].

Even though high-resolution fMRI protocols based on either single-shot EPI or parallel EPI have been previously developed in several research centers, our high-resolution imaging method that integrates multi-band interleaved EPI acquisition and MUSE reconstruction is potentially superior for several reasons. First, the geometric distortions of interleaved EPI data are smaller than that in single-shot EPI data, particularly when choosing a large acquisition matrix size for high-resolution imaging. Second, the PSF of interleaved EPI is sharper than that of single-shot EPI as shown in Fig. 2. Third, as compared with the conventional parallel imaging reconstruction, the developed MUSE reconstruction is less susceptible to undesirable noise amplification. Fourth, using the developed multi-band MUSE algorithm, the unstable aliasing artifacts can be effectively removed and thus the quality of high-throughput and high-resolution fMRI can be significantly improved. However, depending on the chosen temporal domain filtering scheme, the multi-band UNFOLD technique may not completely remove the aliasing artifacts related to shot-to-shot phase variations.

In the implemented two-band interleaved EPI pulse sequence (Fig. 1), the polarity of the second RF pulse alternates between two segments as in reported the CAIPIRINHA technique [13], resulting in FOV shift along the phase-encoding direction and thus improving in matrix inversion conditioning for both MUSE and (through-plane) SENSE reconstruction. This scheme can be extended to different numbers of simultaneously excited slices, through applying appropriate phase modulations to each of the excited slices. It should be pointed out that, instead of using two sequential RF pulses as in Fig. 1, one may apply the cosine modulation to a single excitation RF pulse to simultaneously excite multiple distant slices for multi-band imaging [20]. For multi-band imaging using two sequential RF pulses, the TE difference between two excited slices can be compensated by adding an additional pre-phasing gradient, shifting the TE value of the first excited slice by multiples of the echo-spacing time (*m* × ESP). The phase differences associated with different TE values can be properly estimated in calibration scans, and the information can be used in the multi-band MUSE reconstruction framework. It should be pointed out that the original interleaved EPI acquisition method requires echo-time shifting between shots to ensure smooth signal changes across phase encoding lines. In theory, the echo-time shifting scheme is not needed when the interleaved EPI data are reconstructed by the MUSE algorithm, which assumes that the magnitude proton density maps are consistent across multiple EPI shots. The echo-time shifting scheme was still implemented in out interleaved EPI sequence because our data underwent both conventional interleaved EPI reconstruction and the MUSE reconstruction, for comparison of image quality. Another advantage of the MUSE reconstruction is the flexibility of choosing either full or partial k-space data set as the input. For example, one my use only two segments of a 4-shot interleaved EPI data set in the MUSE reconstruction, to achieve higher temporal resolution at the expense of SFNR reduction.

There are limitations in the currently implemented method. First, the number of EPI segments and number of multi-band slices need to be smaller than the number of RF coil elements oriented along the phase encoding direction and the slice direction, respectively, otherwise the motion-induced phase errors may not be estimated initially with the conventional SENSE method (i.e., step 1 of the MUSE algorithm: see materials and methods section). Second, the positions of eyeballs may change significantly during interleaved EPI acquisition, potentially resulting in residual artifacts in MUSE-produced images. Residual aliasing artifacts related to eyeball movement can be eliminated by placing a spatial saturation band to suppress the eyeball signals during acquisition [21]. Third, the alterations of time domain signal spectrum and noise correlation pattern resulting from sliding-window averaging were not investigated in this study, since the focus of the current paper is on artifact removal for multi-shot EPI based fMRI due to shot-to-shot signal variation. We would like to point out that the developed multi-band MUSE algorithm can be applied to multi-shot fMRI reconstruction either with or without sliding window averaging. Although the incorporation of sliding-window averaging into the multi-shot fMRI reconstruction can produce more data time points, its impact on the time domain noise correlation should be further investigated in future studies.

In conclusion, the techniques reported in this paper enable interleaved EPI based fMRI of significantly improved quality, and should be highly valuable for high-resolution fMRI studies. The generalized MUSE algorithm can accommodate multi-band interleaved EPI data, effectively and simultaneously eliminating both in-plane and through-plane aliasing artifacts in high-throughput and high-resolution fMRI data.
